# Supplementary material for: Bridging the gap in customised housing design: Integrating a graphic user interface for user collaboration
Source: PLoS One. 2024 Dec 20;19(12):e0313291. doi: 10.1371/journal.pone.0313291 (PMC11661643; doi:10.1371/journal.pone.0313291)
Supplement: S1 File — (PDF) [file pone.0313291.s001.pdf]

(Portuguese Version)

QUESTIONÁRIO A PROFISSIONAIS

1 – Profissionais com experiência em processos participativos

1. Acha que o fato de um edifício (ou conjunto de edifícios) ser projetado para habitação em massa, dificulta a capacidade de desenhar de modo personalizado?

2. Acha que para uma habitação ser personalizada é essencial que o utilizador final participe no processo de projeto?

3. Como ocorreu a participação?
   a. Em que aspetos os habitantes participaram?

4. Qual considera ser a melhor forma de lidar com um grupo de pessoas num processo participativo?

5. Quais foram as dificuldades ou limitações no processo?

6. Sentiu que a comunidade tinha interesse em participar?

7. Quantas pessoas participaram? O grupo era variado (relativamente à idade, profissão, capacidade de perceção do projeto…)?

8. Acha que os habitantes devem “pôr mãos à obra”, colaborando no desenho e não apenas na tomada de decisões?
   a. Como podem eles colaborar no desenho?
   b. Quais seriam os limites dessa colaboração?

9. O que foi apresentado aos participantes? Em que suporte?

10. O que os participantes fizeram?

11. Considera que foi um processo de co-design ou de participação?

12. Foi utilizado algum tipo de tecnologia digital? Qual?

13. De que forma as tecnologias digitais podem auxiliar estes processos?

14. De que formas as tecnologias influenciam a perceção do espaço por não profissionais?

15. De que forma as tecnologias podem envolver/ entusiasmar a comunidade?

16. De que forma as tecnologias podem balancear entre as limitações dos regulamentos de construção e a liberdade da comunidade desenhar?

17. Como pode um grupo de pessoas colaborar usando a mesma ferramenta digital (e em simultâneo)?

2 – Profissionais sem experiência em processos participativos

1. Acha que as habitações inseridas em edifícios ou bairros de habitação em massa devem ser personalizadas, isto é, ajustadas às necessidades individuais dos seus habitantes?

2. Acha que o fato de um edifício (ou conjunto de edifícios) ser projetado para habitação em massa, dificulta a capacidade de desenhar de modo personalizado?

3. Acha que para uma habitação ser personalizada é essencial que o utilizador final participe no processo de projeto?
   a. Em que moldes considera que esta participação pode ocorrer?

4. Quais considera ser as consequências da participação dos habitantes no projeto da sua casa?

5. Qual considera ser a melhor forma de lidar com um grupo de pessoas num processo participativo?

6. Quais são as dificuldades que causam a falta de processos participativos em arquitetura?

7. Considera que a comunidade tem interesse em participar?

8. Acha que os habitantes devem "pôr mãos à obra", colaborando no desenho e não apenas na tomada de decisões?
   a. Como podem eles colaborar no desenho?
   b. Quais seriam os limites dessa colaboração?

9. Que métodos são mais eficazes para conduzir um processo participativo?

10. De que forma as tecnologias digitais podem facilitar estes processos?

(English Version)

QUESTIONNAIRE FOR PROFESSIONALS

1 – Group A: Professionals with experience in participatory processes

1. Do you think that the fact that a building (or set of buildings) is designed for mass housing hinders the ability to design in a customised way?

2. Do you think that the end user's participation in the design process is essential for a house to be customised?

3. How did the participation occur?
   a. In what aspects did the community participate?

4. What is the best way to deal with a group of people in a participatory process?

5. What were the difficulties or limitations in the process?

6. Did you feel that the community was interested in participating?

7. How many people participated? Was the group diversified (in terms of age, profession, perception of the design…)?

8. Do you consider that the inhabitants should “put their hands on” the design, collaborating on it rather than just in decision-making?
   a. How can they collaborate in the design?
   b. What would be the limits of this collaboration?

9. What was presented to the participants? On what support?

10. What did the participants do?

11. Do you consider it was a process of co-design or participation?

12. Was any type of digital technologies used? What type of digital technology was used?

13. How can digital technologies assist these processes?

14. How do digital technologies influence non-designers' perception of space?

15. How can digital technologies engage the community?

16. How can digital tools balance the limitations of construction regulations and the freedom of the community to design?

17. How can a group of people collaborate simultaneously using the same digital tool?

2 – Group B: Professionals without experience in participatory processes

1. Do you think houses within mass housing projects should be customised, that is, adjusted to the individual needs of their inhabitants?

2. Do you think that the fact that a building (or set of buildings) is designed for mass housing hinders the ability to design in a customised way?

3. Do you think that the end user's participation in the design process is essential for a house to be customised?
   a. In what ways do you think this participation can occur?

4. What are the consequences of the inhabitants' participation in the design of their houses?

5. What is the best way to deal with a group of people in a participatory process?

6. What do you consider to be the difficulties that cause the few participatory processes in architecture?

7. Do you consider the community is interested in participating in participatory processes?

8. Do you consider that the inhabitants should “put their hands on” the design, collaborating on it rather than just on decision-making?
   a. How can they collaborate in the design?
   b. What would be the limits of this collaboration?

9. What are the most effective methods for conducting a participatory process?

10. How can digital technologies assist these processes?

(Original Version)

QUESTIONÁRIO A COOPERATIVAS

1. Como funciona na associação/cooperativa o processo de construção de edifícios de habitação – quem são os interlocutores e quais são as fases?

2. Sobre os edifícios de habitação coletiva que construíram:
   - **a.** Em que zonas geográficas têm trabalhado mais?
   - **b.** Para que níveis económicos da população têm trabalhado mais?
   - **c.** De que faixas etárias são os associados da cooperativa?

3. Nos edifícios de habitação que promovem, o projeto de arquitetura é realizado por um escritório de arquitetura por vocês contratado? É normal contratar várias vezes o mesmo escritório?

4. Houve participação dos habitantes/ cooperadores no processo de desenho das suas casas?
   - **a.** Quem impulsionou a participação dos habitantes? (habitantes, cooperativa, arquiteto…)
   - **b.** Em que momentos os habitantes intervieram?
   - **c.** De que forma participaram? Como foi dinamizada a sessão de participação?
   - **d.** Os habitantes mostraram-se motivados em participar? (senão, como é que foi feito?)

5. (Se não houve participação) Os habitantes expressaram a opinião de que gostariam de ter participado?

6. Qual era a diversidade do grupo de habitantes que participou nas sessões relativamente à idade, profissão, capacidade de perceção do projeto, ligação com tecnologias, etc?

7. Acha que os habitantes ficaram satisfeitos com o resultado?

(Translated Version)

QUESTIONNAIRE FOR HOUSING COOPERATIVES

1. How does the process of building housing in the association/cooperative – who are the stakeholders, and what are the phases?

2. About the collective housing buildings the cooperative built:
   - **a.** In which geographic areas have you worked the most?
   - **b.** For which economic levels of the population have you worked the most?
   - **c.** What age groups are the cooperative members from?

3. In the residential buildings you promote, is the architectural design carried out by an architectural office you hire? Is it normal to hire the same office several times?

4. Was there participation of the inhabitants/cooperators in the process of designing their houses?
   - **a.** Who encouraged the participation of the inhabitants? (inhabitants, cooperative, architect…)
   - **b.** When did the inhabitants step in?
   - **c.** How did they participate? How was the participation session organised?
   - **d.** Were the inhabitants motivated to participate? (If not, how was it done?)

5. (If there was no participation) Did the inhabitants express the opinion that they would have liked to have participated?

6. What was the diversity of the group of residents who participated in the sessions in terms of age, profession, ability to understand the project, relationship with technologies, etc?

7. Do you think the inhabitants were satisfied with the result?

(Original Version)

QUESTIONÁRIO A HABITANTES

1 – Introdução – Criar empatia

1. Como se chama?
2. Que idade tem?
3. Qual é o seu nível de escolaridade?
4. Qual é a sua profissão?
5. O que gosta de fazer?
6. O que é mais importante para si em relação à sua nova casa (o que gostaria mais que a casa tivesse / O que mais gosta na sua casa)? Porquê?
7. Se pudesse mudar alguma coisa na sua casa, o que seria?
8. Como é a sua relação com as tecnologias digitais?
    a. Que tecnologias usa diariamente?
        i. Quais os dispositivos e para que atividades

2 – Perguntas exploratórias – Sobre o processo que passou

9. Porque procurou uma cooperativa para fazer a sua casa? (Motivação – O que queria?)
10. Quando foi o projeto?
11. Quem participou? (perguntar sobre outros intervenientes, ex: cooperativa, arquiteto…)
12. Sobre que partes do projeto deu a sua opinião ou ideias, e como se decidiram os espaços comuns (se aplicável)?
13. Como participou - o que fez exatamente e com que materiais?
14. O que lhe foi mostrado pelo arquiteto de modo a perceber o projeto?
15. Que dificuldades sentiu? Sentiu dificuldades em entender o que lhe foi mostrado?
16. Que diferenças houve entre a sua perceção inicial da casa, em projeto, e o resultado final construído?

3 – Conclusão – Sobre satisfação (Pains/Gains)

17. Ficou satisfeito com o resultado (do projeto) da sua casa? O resultado foi idêntico ao que esperava?

- **a.** Acha que se (não) tivesse participado, o resultado seria o mesmo?

18. O que mudaria agora e porquê?

19. Qual foi o aspeto do processo que mais gostou?

20. O que gostaria que tivesse sido diferente no processo?

- **a.** Na sua relação com o projetista
- **b.** No número de vezes que falou com o responsável pelo projeto
- **c.** Nos elementos com os quais trabalhou - o que gostaria de ter feito que não fez ou vice-versa (Desenhar, manipular objetos, falar, escrever, representar…- com objetos físicos ou digitais)

21. Gostaria que o projetista acompanhasse o processo integralmente ou preferia explorar sozinho (ou com família, amigos…) partes do projeto? Porquê?

22. Escolha que elementos gostaria que um sistema digital tivesse, para o ajudar a participar no projeto da sua casa. Ordene-os por prioridade.

- **a.** Elementos de arquitetura

Elementos que gostaria de poder decidir		Prioridade
Forma da casa		
Quantidade e dimensão de cada tipo de divisão		
Layout da habitação inteira (automático)		
Layout de cada divisão (passo a passo)		
Organização do interior (posição e relação entre divisões)		
Limites e permeabilidades		
Materiais e acabamentos		

b. Tipo de interação

Elementos que gostaria de poder decidir		Prioridade
Drag and Drop		
Clicar em imagens/botões		
Desenhar		
Escolha tipo catálogo		
Sliders		
Preenchimento de campos		

c. Tipo de visualização

Elementos que gostaria de poder decidir		Prioridade
Planta/ desenhos esquemáticos		
3D esquemático		
Planta/ desenhos técnicos detalhados		
Planta/ desenhos animados		
Vistas interiores estática		
3D interativo/ walkthrough		

d. Dispositivos e interface

Elementos que gostaria de poder decidir		Prioridade
Computador (rato e teclado)		
Smartphone (touchscreen)		
Tablet (touchscreen)		
Mesa digital (multi-touchscreen)		
HMD (realidade virtual imersiva)		

(Translated Version)

QUESTIONNAIRE FOR INHABITANTS

1 – Introduction – Creating empathy

1. What is your name?
2. How old are you?
3. What is your level of education?
4. What is your profession?
5. What do you like to do?
6. What is most important to you about your new home (what would you like the house to have / What do you like most about your home)? Why?
7. What would it be if you could change anything in your home?
8. How is your relationship with digital technologies?
    a. What technologies do you use daily?
        i. Which devices and for what activities

2 – Exploratory questions – About the process they went through

9. Why did you look for a cooperative to build your house? (Motivation – What did they want?)
10. When was the project?
11. Who was involved? (ask about other stakeholders, e.g., cooperative, architect…)
12. About which parts of the project did you give your opinion or ideas, and how were the common spaces decided (if applicable)?
13. How did you participate – what exactly did you do, and with what materials?
14. What was shown to you by the architect in order to understand the design?
15. What difficulties did you experience? Did you have difficulty understanding what was shown to you?
16. What differences were there between your initial perception of the house, as a design, and the final result that was built?

3 – Conclusion – About satisfaction (Pains/Gains)

17. Were you satisfied with your house's result (of the design)? Was the result identical to what you expected?

   a. Do you think that if you had (not) participated, the result would have been the same?

18. What would you change now and why?

19. What was the aspect of the process you enjoyed the most?

20. What would you like to have been different in the process?

   a. Regarding your relationship with the designer

   b. Regarding the number of times you spoke to the person responsible for the project

   c. Regarding the elements you worked with - what would you have liked to have done that you did not do or vice versa (Drawing, manipulating objects, talking, writing, representing... - with physical or digital objects)

21. Would you like the designer to follow the entire process, or would you prefer to explore parts of the project alone (or with family, friends...)? Why?

22. Decide which elements you would like a digital system to have to help you participate in the design of your house. Order them by priority.

   a. Features

Elements they wish they could decide		Priority
House shape		
Rooms number and dimensions		
Automatic generation of the entire house’s layout		
Step by step layout		
Interior layout arrangement		
Limits and permeabilities		
Finishing materials		

b. Type of interaction

Elements they wish they could decide		Priority
Drag and Drop		
Clicking on images or buttons		
To Draw		
Choosing from a catalogue		
Sliders		
Field filling		

c. Type of visualisation

Elements they wish they could decide		Priority
Schematic drawings		
Schematic three-dimensional (3D) model		
Detailed drawings		
Humanised floorplans		
Static interior views		
Interactive/walkthrough 3D model		

d. Devices

Elements they wish they could decide		Priority
Computer (mouse and keyboard)		
Smartphone (touchscreen)		
Tablet (touchscreen)		
Interactive digital table (multi-touchscreen)		
HMD (immersive Virtual Reality)		
