# Supplementary material for: Bridging the gap in customised housing design: Integrating a graphic user interface for user collaboration
Source: PLoS One. 2024 Dec 20;19(12):e0313291. doi: 10.1371/journal.pone.0313291 (PMC11661643; doi:10.1371/journal.pone.0313291)

Consolidation of usability problems

Severity	Priority
**3**	**P1- More frequent and severe**
Warnings	**(3x)** Library options' caption
Irreversible action information	**(2x)** Order of associating the material with the element
Undo/Redo	**(2x)** Drag the material
"Customise" button	**(2x)** Warnings
Accept/cancel new solution	**(2x)** Order of hiding walls in the 3D view
3D model menu options	**P2- Severe non-frequent**
Order of hiding walls in the 3D view	Undo/Redo
Order of associating the material with the element	Applying the material
Applying the material	Irreversible action information
Scroll	3D model menu options
Library options' caption	Accept/cancel new solution
Order of changing the doors	Order of changing the doors
Drag the material	"Customise" button
**2**	Scroll
Start	**P3- Frequent non-severe**
Help	
Home and Back buttons	**(2x)** "My projects"
Cancel button on the customise room option	**P4- Non-severe nor frequent**
Identification of rooms on the floorplan	Start
Logo	Help
"Hide" icon	Home and Back buttons
"My projects"	Identification of rooms on the floorplan
"Hide" button denomination	"Hide" button denomination
**1**	Cancel button on the customise room option
Icons in "my projects" interface	Logo
"My home"	"Hide" icon
Apartment identification	**P5- Do not need to be resolved**
Number of occupants	Icons in "my projects" interface
"Choices made" identification	"My home"
3D orthographic views	Number of occupants
"Continue customising" button	"Choices made" identification
Materials denomination	Apartment identification
Delete walls	"Continue customising" button
Windows	3D orthographic views
	Materials denomination
	Delete walls
	Windows

Warnings

PROBLEM DESIGNATION: Warnings

HEURISTICS VIOLATED: 5- Error Prevention (Nielsen's Heuristics)

PROBLEM DESCRIPTION: When confirmation warnings appear about important and non-reversible actions, the button that draws the most attention (the one highlighted in dark purple) is "Accept". Since the user may not pay attention to the text of the warning and has a tendency to click on the highlighted button, such a button should be the "Cancel" button to prevent the user from accidentally committing to an action that he did not intend.

SOLUTION PROPOSAL: Assign dark purple to the "Cancel" button instead of the "Accept" as it currently is.

SEVERITY DEGREE: 3

Image of the interface

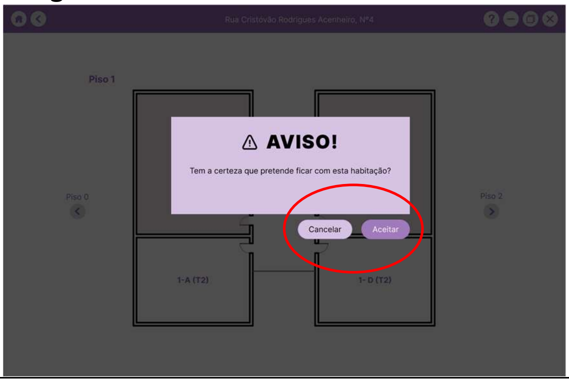

Irreversible action information

PROBLEM DESIGNATION: Irreversible action information

HEURISTICS VIOLATED: 5- Error Prevention (Nielsen's Heuristics)

PROBLEM DESCRIPTION: The warning does not indicate that the action is irreversible. The text of the warning must indicate this information so that the user is aware of the consequences of their decisions.

SOLUTION PROPOSAL: Add in the warning the information that when choosing a house, the user cannot change that decision later.

SEVERITY DEGREE: 3

Image of the interface

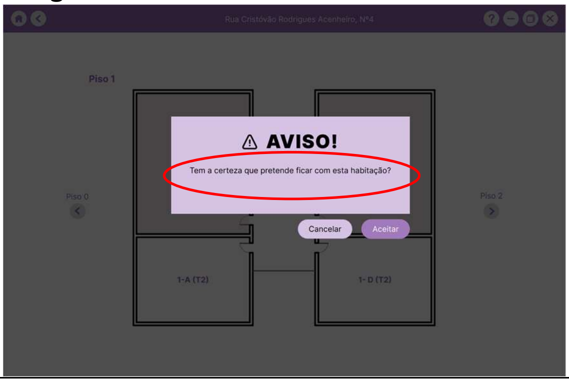

Undo/Redo

**PROBLEM DESIGNATION:** Undo/Redo
**HEURISTICS VIOLATED:** 4- Consistency and standards (Nielsen's Heuristics)
**PROBLEM DESCRIPTION:** Different features cannot have similar appearance - The Undo icon is the same as navigating backwards in previous stages.<br>In addition to being the same, it is not consistent with other platforms that have the same functionality, not representing its function well
**SOLUTION PROPOSAL:** Change the Undo/Redo icons
**SEVERITY DEGREE:** 3
**Image of the interface**

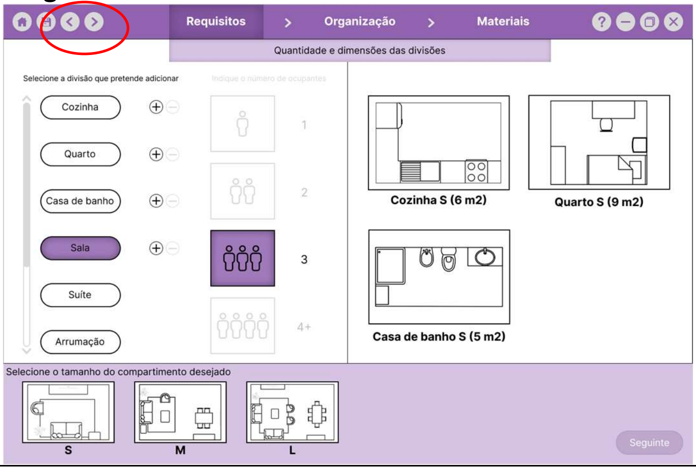

"Customise" button

**PROBLEM DESIGNATION: "**Customise" button
**HEURISTICS VIOLATED:** 4- Consistency and standards (Nielsen's Heuristics)
**PROBLEM DESCRIPTION:** Different functionalities cannot have similar appearances: Customise must not have the same appearance as the previous buttons, because it is intended to create a new label
**SOLUTION PROPOSAL:** Replace the name "personalizar" (Customise), for example, with a "+" so that it does not look like a button, like the previous ones
**SEVERITY DEGREE:** 3
**Image of the interface**

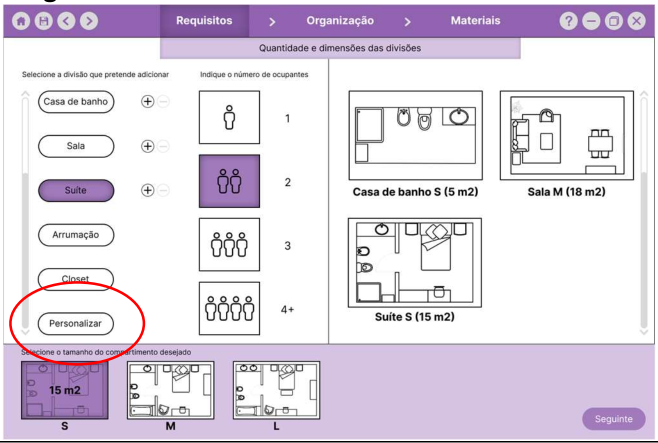

Accept/cancel new solution

**PROBLEM DESIGNATION:** Accept/cancel new solution
**HEURISTICS VIOLATED:** 2- Match between the system and the real world (Nielsen's Heuristics)
**PROBLEM DESCRIPTION:** When the warning appears, when changing the room with the suite, there are the options to cancel, generate a new solution, or accept. "Accept" means accepting the proposed solution (the design cannot be changed as the user changed it because it does not comply with the regulations), and "cancel" means cancelling the change that was made (exchanging the room with the suite) – This information is not clear with the buttons just saying "accept" and "cancel"<br>The generate new solution button is also not clear about what it does and should not be grouped with the accept and cancel buttons
**SOLUTION PROPOSAL:** Change the name of the buttons to "accept proposed solution", "cancel change" and "generate alternative", and place the "generate alternative" button within the frame, below the solution
**SEVERITY DEGREE:** 3
**Image of the interface** 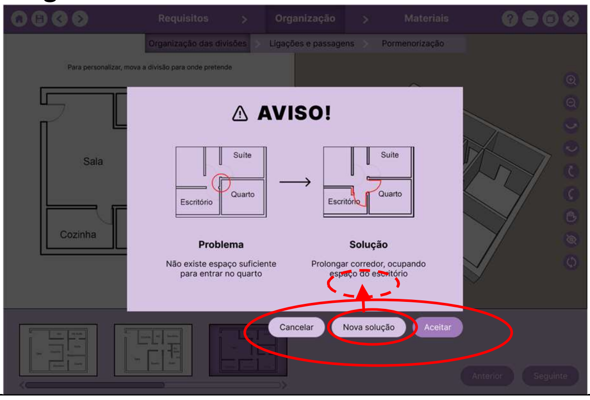

3D model menu options

**PROBLEM DESIGNATION:** 3D model menu options
**HEURISTICS VIOLATED:** 1- Visibility of the system status (Nielsen's Heuristics)
**PROBLEM DESCRIPTION:** The system must indicate that something has happened. In 3D, when the user wants to hide the wall, when he/she clicks on the "hide" button it changes colour (it becomes darker purple as it is selected) - this change is very subtle and the user does not notice that it has changed colour.
**SOLUTION PROPOSAL:** Intensify the button's state change, for example, making the line around it thicker
**SEVERITY DEGREE:** 3
**Image of the interface** 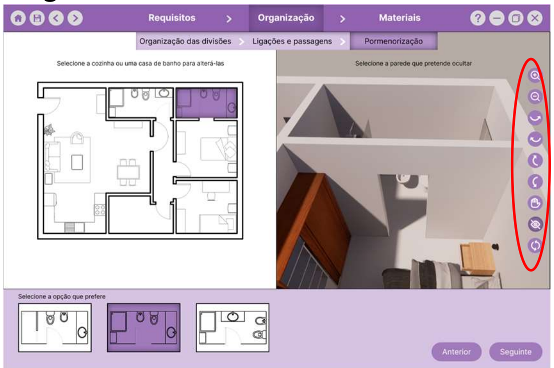

Order of hiding walls in the 3D view

**PROBLEM DESIGNATION:** Order of hiding walls in the 3D view
**HEURISTICS VIOLATED:** 7- Flexibility and efficiency of use (Nielsen's Heuristics)
**PROBLEM DESCRIPTION:** In *Detailing* (Pormenorização), some people tend to first choose the wall and then say what they want to do with it (hide it in 3D). On the other hand, some people do it the other way around (first click the *Hide* button and then select the wall they want to hide). Doing it in just one of the ways may make some users not know how to act.
**SOLUTION PROPOSAL:** The system should give the option to do it both ways, or the wall should automatically disappear (see the wall disappearing), because it is not mandatory to change the view, only if the user wants to see it better
**SEVERITY DEGREE:** 3
**Image of the interface** 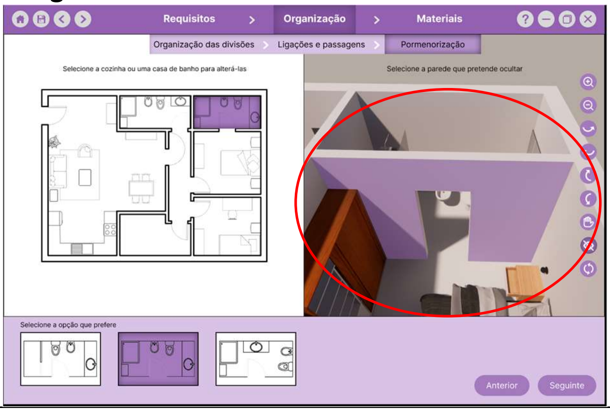

Order of associating the material with the element

**PROBLEM DESIGNATION:** Order of associating the material with the element
**HEURISTICS VIOLATED:** 7- Flexibility and efficiency of use (Nielsen's Heuristics)
**PROBLEM DESCRIPTION:** In *Materials*, the task is to choose a material for a room/element. By first choosing the material, the user considers the task complete because they have achieved the goal. However, it still needs to be associated with the room/design element.
**SOLUTION PROPOSAL:** Change the sequence of actions when choosing materials - first choose the room/design element where to place the material and then choose the material. Or allow both ways
**SEVERITY DEGREE:** 3
**Image of the interface** 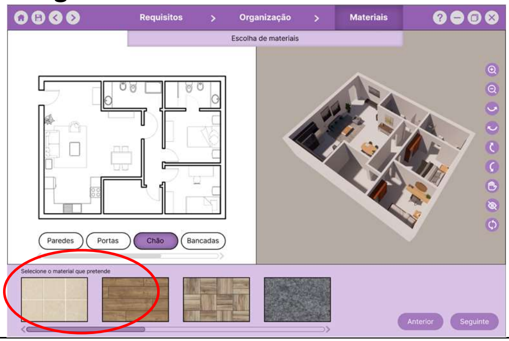

Applying the material

PROBLEM DESIGNATION: Applying the material

HEURISTICS VIOLATED: **(1)** 5- Error Prevention (Nielsen's Heuristics); **(2)** 8- Aesthetic and minimalist design (Nielsen's Heuristics)

PROBLEM DESCRIPTION: **(1)** It's not a good practice to drag something from the primary menu because it gives access to a set of options. If the user drags from this menu, they do not know which option they are assigning to the room. It must be dragged from the menu where the material option that the user chose is located. However, in this case, it cannot be dragged either because it is a popup menu and therefore is placed in front of the floorplan. **(2)** Furthermore, the number of functions per control must be minimised - the ideal would be one function per control - in this case, the material buttons have two functions: 1- click to open the pop-up menu and choose the option, and 2- drag to assign the material to the room.

SOLUTION PROPOSAL: Change the order in which the material is applied, i.e, first choose where the material will be applied and then select the material option (without dragging). Thus, when selected, the material is automatically associated with the previously chosen room/element.

SEVERITY DEGREE: 3

Image of the interface

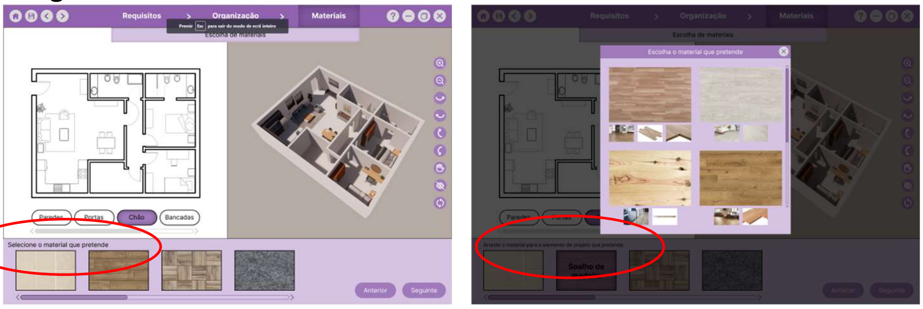

Scroll

PROBLEM DESIGNATION: Scroll

HEURISTICS VIOLATED: 1- Visibility of the system status (Nielsen's Heuristics)

PROBLEM DESCRIPTION: When functions are hidden, it is difficult for the user to discover and know how to use the interface. Scrolling should be avoided.

SOLUTION PROPOSAL: Divide information across more screens.

SEVERITY DEGREE: 3

Image of the interface

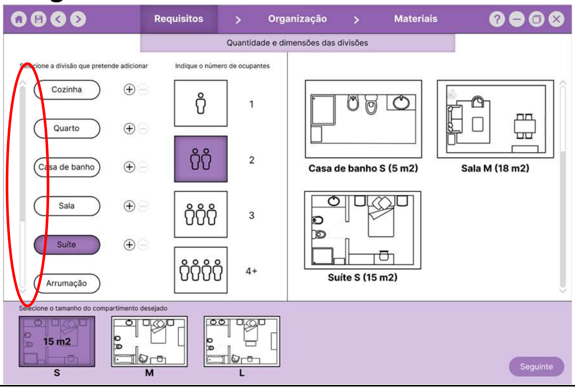

Library options' caption

**PROBLEM DESIGNATION:** Library options' caption
**HEURISTICS VIOLATED:** 6- Recognition rather than recall (Nielsen's Heuristics)
**PROBLEM DESCRIPTION:** Options are not identified. When sliding the scroll bar, what was the 3rd option is no longer in the 3rd position. The user should not have to memorise the options
**SOLUTION PROPOSAL:** Caption options
**SEVERITY DEGREE:** 3
**Image of the interface** 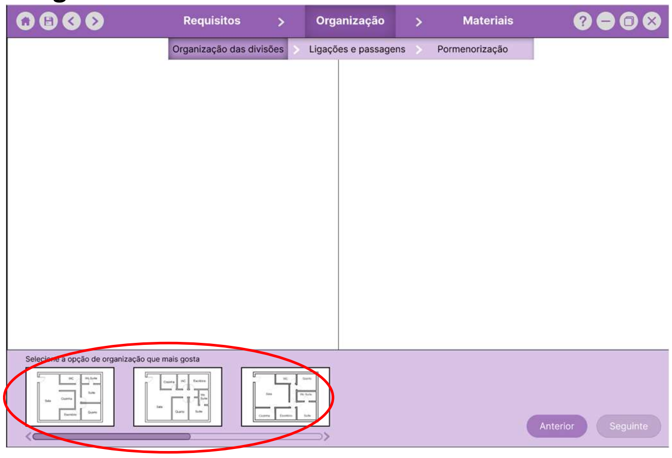

Order of changing the doors

**PROBLEM DESIGNATION:** Order of changing the doors
**HEURISTICS VIOLATED:** 7- Flexibility and efficiency of use (Nielsen's Heuristics)
**PROBLEM DESCRIPTION:** In the connections and passages task, some people will first click on the "change doors" button, but there may also be a tendency to click on the door directly before selecting the button.
**SOLUTION PROPOSAL:** Allow both options – if the user clicks on the door directly, the system activates the button automatically
**SEVERITY DEGREE:** 3
**Image of the interface** 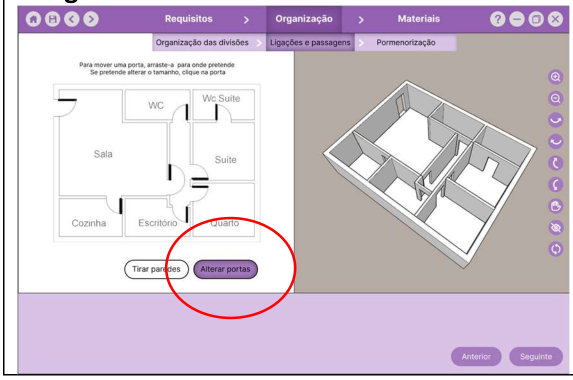

Drag the material

PROBLEM DESIGNATION: Drag the material

HEURISTICS VIOLATED: 1- Visibility of the system status (Nielsen’s Heuristics)

PROBLEM DESCRIPTION: The sentence that tells the user to drag the material to the desired room, despite pulsing, does not stand out.

SOLUTION PROPOSAL: The sentence should be in another area of the interface (higher up), and highlighted (with bold or another colour that attracts attention)

SEVERITY DEGREE: 3

Image of the interface

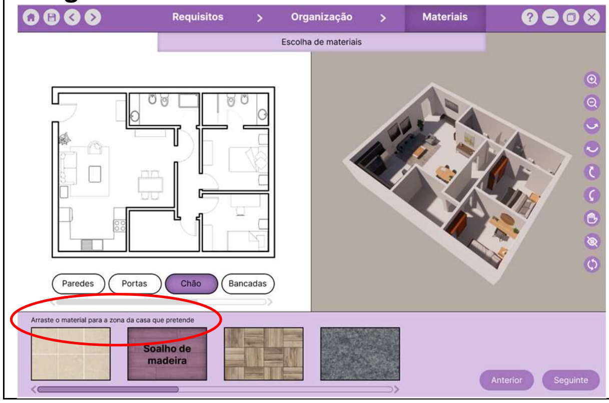

Start

PROBLEM DESIGNATION: Start

HEURISTICS VIOLATED: 1- Visibility of the system status (Nielsen’s Heuristics)

PROBLEM DESCRIPTION: Right on the start, the user may think that the system is processing. There is no element that indicates the state of the system.

SOLUTION PROPOSAL: There should be a “start” button or something similar

SEVERITY DEGREE: 2

Image of the interface

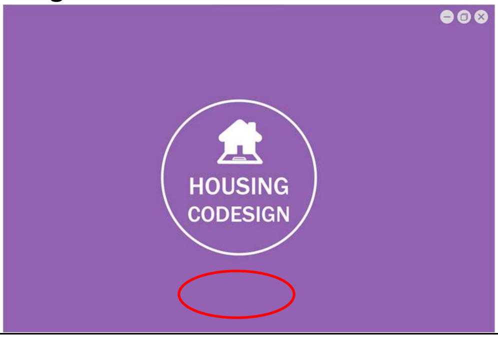

Help

**PROBLEM DESIGNATION:** Help
**HEURISTICS VIOLATED:** 4- Consistency and standards (Nielsen's Heuristics)
**PROBLEM DESCRIPTION:** In *My Projects*, there is a help tab and the help button (next to close, minimise and maximise) - it is redundant to have two helps
**SOLUTION PROPOSAL:** Remove the "Help" tab from the side menu
**SEVERITY DEGREE:** 2
**Image of the interface**

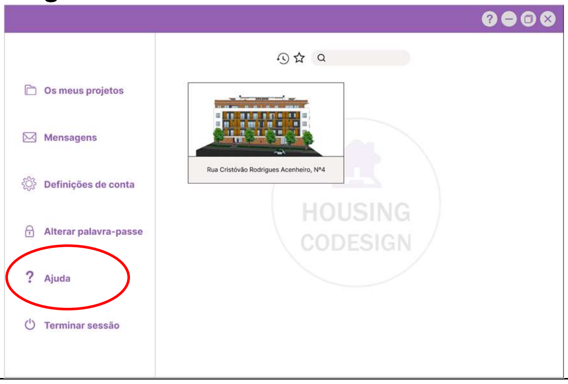

Home and Back buttons

**PROBLEM DESIGNATION:** Home and Back buttons
**HEURISTICS VIOLATED:** 4- Consistency and standards (Nielsen's Heuristics)
**PROBLEM DESCRIPTION:** Having "Home" and "Back" buttons is redundant because they will lead to the same place
**SOLUTION PROPOSAL:** Remove the "Home" button
**SEVERITY DEGREE:** 2
**Image of the interface**

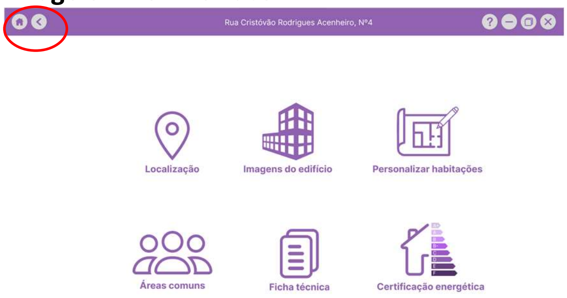

Cancel button on the customise room option

**PROBLEM DESIGNATION:** Cancel button on the customise room option
**HEURISTICS VIOLATED:** 3- User control and freedom (Nielsen's Heuristics)
**PROBLEM DESCRIPTION:** There is no option to cancel entering a custom room. It's not too serious because the user can delete the text they entered to name the room or click anywhere else in the interface, and the entry is cancelled.
**SOLUTION PROPOSAL:** Add a "Cancel" near "Ok" (does not need to be "Cancel" and "Ok", it can be a "√" and "X")
**SEVERITY DEGREE:** 2
**Image of the interface**

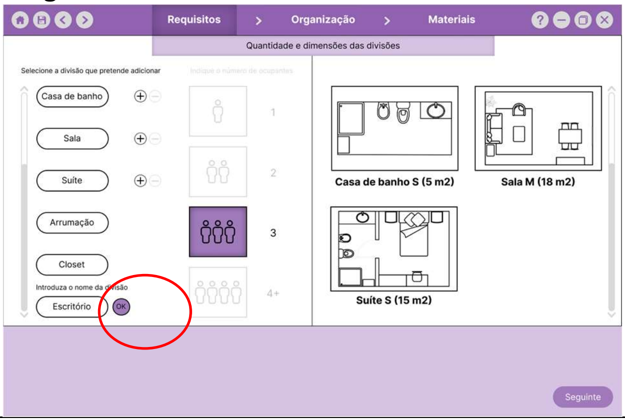

Identification of rooms on the floorplan

**PROBLEM DESIGNATION:** Identification of rooms on the floorplan
**HEURISTICS VIOLATED:** 6- Recognition rather than recall (Nielsen's Heuristics)
**PROBLEM DESCRIPTION:** In the detail phase, hovering the cursor over the rooms highlights them (on the floorplan). The room's name should also appear because the user should not have to remember where the kitchen was. If it is identified it is easier to recognise the room
**SOLUTION PROPOSAL:** Caption spaces on the floorplan when hovering the cursor over them
**SEVERITY DEGREE:** 2
**Image of the interface**

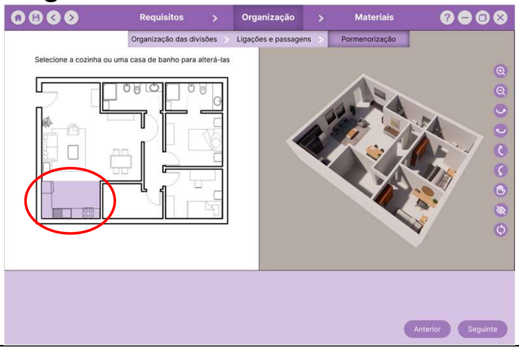

Logo

PROBLEM DESIGNATION: Logo

HEURISTICS VIOLATED: 2- Match between the system and the real world (Nielsen's Heuristics)

PROBLEM DESCRIPTION: The system must use the user's language. If the user is Portuguese, the logo in English can make participants feel withdrawn as they do not feel comfortable with English, and think that the entire interface will be in English.

SOLUTION PROPOSAL: Change to Portuguese

SEVERITY DEGREE: 2

Image of the interface

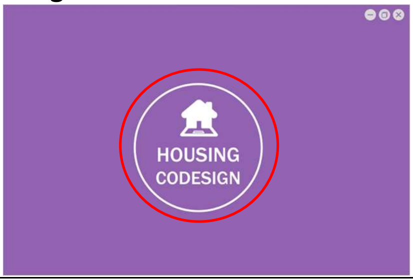

"Hide" icon

PROBLEM DESIGNATION: "Hide" icon

HEURISTICS VIOLATED: 2- Match between the system and the real world (Nielsen's Heuristics)

PROBLEM DESCRIPTION: The system must use the user's language. The wall hide button icon may not be recognised by the user. If the expert did not recognise it, it is possible that the user will not recognise it either. However, when hovering the cursor over it, the denomination of the button appears.

SOLUTION PROPOSAL: Change the icon so that it is clearer to the user

SEVERITY DEGREE: 2

Image of the interface

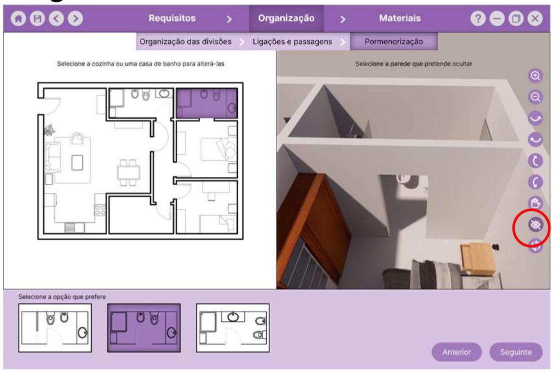

“My projects”

**PROBLEM DESIGNATION:** “My projects”
**HEURISTICS VIOLATED:** 3- User control and freedom (Nielsen’s Heuristics)
**PROBLEM DESCRIPTION:** In “my projects” there should be more options.
**SOLUTION PROPOSAL:** Add more projects options to choose from.
**SEVERITY DEGREE:** 2
**Image of the interface** 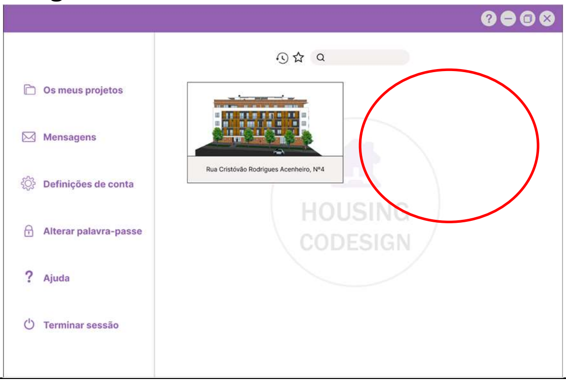

“Hide” button denomination

**PROBLEM DESIGNATION:** “Hide” button denomination
**HEURISTICS VIOLATED:** 1- Visibility of the system status (Nielsen’s Heuristics)
**PROBLEM DESCRIPTION:** If the user hovers the cursor over the “hide” button when it is selected, the button designation does not appear. This may confuse the user, as they may not remember the button’s function.
**SOLUTION PROPOSAL:** Add the button name similarly to what occurs with all other buttons in the 3D menu
**SEVERITY DEGREE:** 2
**Image of the interface** 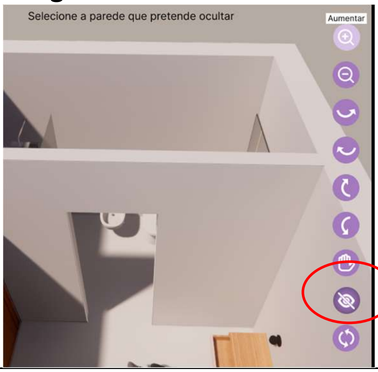

Icons in “my projects” interface

**PROBLEM DESIGNATION:** Icons in “my projects” interface
**HEURISTICS VIOLATED:** 8- Aesthetic and minimalist design (Nielsen’s Heuristics)
**PROBLEM DESCRIPTION:** The icons concentrated in the center should be in another position
**SOLUTION PROPOSAL:** Position the icon group in the upper right corner, below the toolbar
**SEVERITY DEGREE:** 1
**Image of the interface** 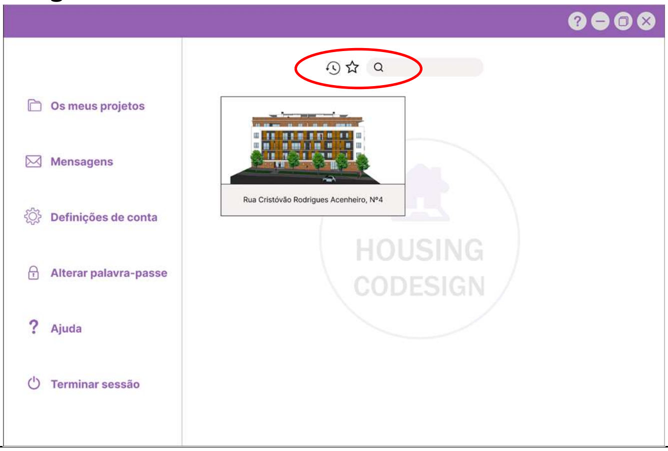

“My home”

**PROBLEM DESIGNATION:** “My home”
**HEURISTICS VIOLATED:** 2- Match between the system and the real world (Nielsen’s Heuristics)
**PROBLEM DESCRIPTION:** After selecting the project, the option “customise houses” should be in the singular (customise house). The greater the evidence of an object, the better the identification of its use. In this case, this option must indicate that the user will only customise their house.
**SOLUTION PROPOSAL:** Change it to “Customize house” or “My house” (so it is not the only option that starts with an action)
**SEVERITY DEGREE:** 1
**Image of the interface** 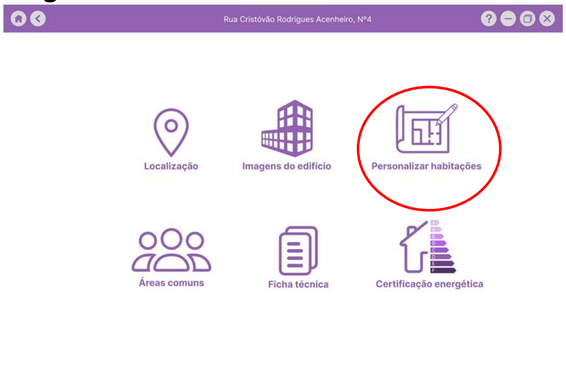

Apartment identification

**PROBLEM DESIGNATION:** Apartment identification
**HEURISTICS VIOLATED:** 6- Recognition rather than recall (Nielsen's Heuristics)
**PROBLEM DESCRIPTION:** When the house has already been chosen and we are already starting to customise the house, it should always be known which apartment has been chosen. The user should not be forced to remember what choice they made previously.
**SOLUTION PROPOSAL:** Add to the interface the identification of the apartment that is being customised (e.g. in the top left corner)
**SEVERITY DEGREE:** 1
**Image of the interface** 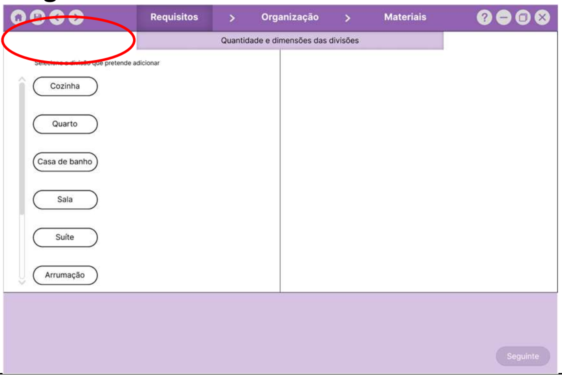

Number of occupants

**PROBLEM DESIGNATION:** Number of occupants
**HEURISTICS VIOLATED:** 1- Visibility of the system status (Nielsen's Heuristics)
**PROBLEM DESCRIPTION:** When choosing requirements, the user may be confused about why the number of occupants is previously selected and blocked. The system must provide this information
**SOLUTION PROPOSAL:** Place an indication that the number of occupants was defined (with the exception of bedrooms) depending on the household (e.g. when hovering the cursor over the button)
**SEVERITY DEGREE:** 1
**Image of the interface** 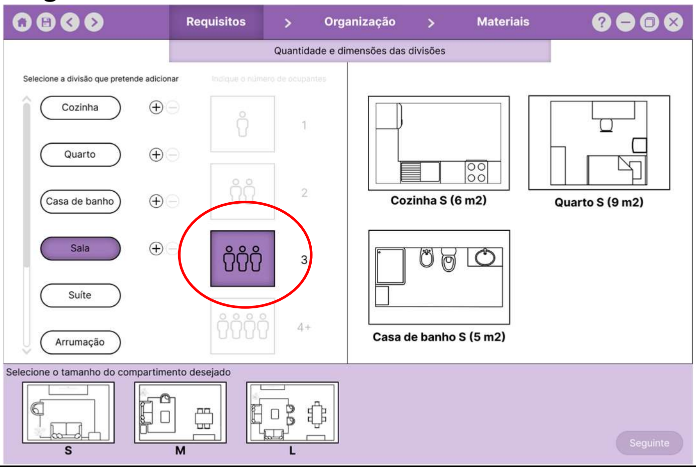

“Choices made” identification

PROBLEM DESIGNATION: “Choices made” identification

HEURISTICS VIOLATED: 1- Visibility of the system status (Nielsen’s Heuristics)

PROBLEM DESCRIPTION: The user may not realise that the workspace on the right screen is where the summary of the choices appears. The system must provide this information

SOLUTION PROPOSAL: Place in the workspace on the right side: “summary of your choices”

SEVERITY DEGREE: 1

Image of the interface

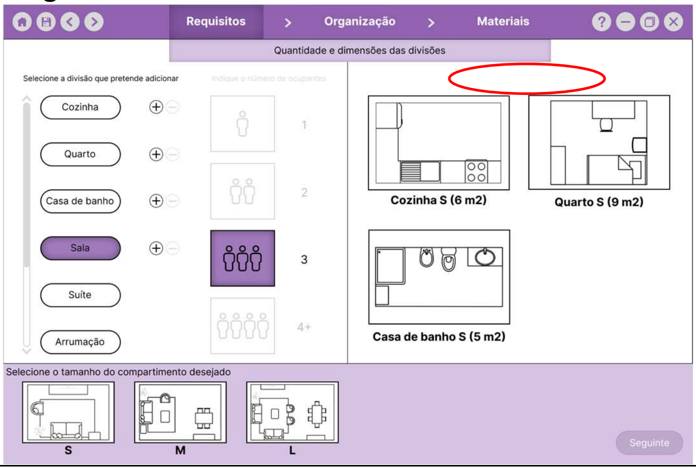

3D orthographic views

PROBLEM DESIGNATION: 3D orthographic views

HEURISTICS VIOLATED: Heuristics are not applied because it is a matter of architecture visualisation.

PROBLEM DESCRIPTION: In the 3D interaction menu, there should also be orthographic views - at least the top view

SOLUTION PROPOSAL: Add options for orthographic views

SEVERITY DEGREE: 1

Image of the interface

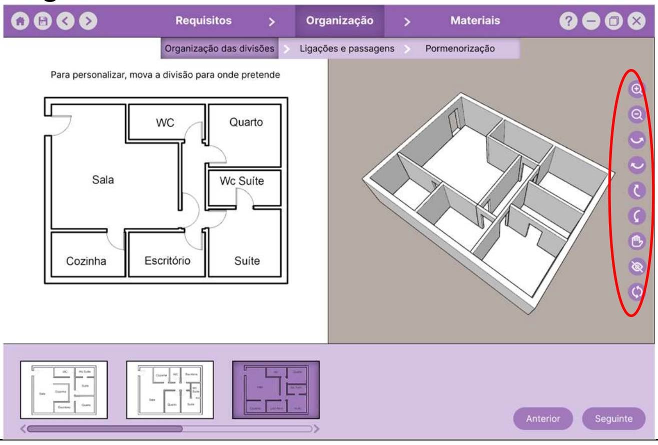

“Continue customising” button

**PROBLEM DESIGNATION:** “Continue customising” button
**HEURISTICS VIOLATED:** 2- Match between the system and the real world (Nielsen’s Heuristics)
**PROBLEM DESCRIPTION:** Interface elements must be clear about what will happen if the user interacts with them. When finishing the project, after having chosen the finishing materials, the “Back” button is not clear about what happens if the user clicks on it.
**SOLUTION PROPOSAL:** Replace the name of the “Back” button with “Continue to customise”
**SEVERITY DEGREE:** 1
**Image of the interface**

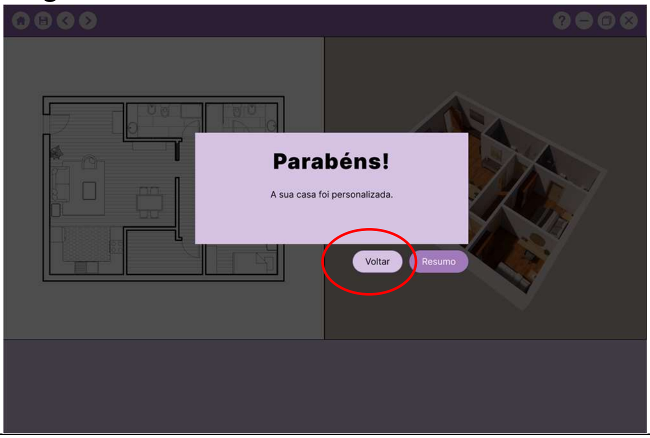

Materials denomination

**PROBLEM DESIGNATION:** Materials denomination
**HEURISTICS VIOLATED:** 2- Match between the system and the real world (Nielsen’s Heuristics)
**PROBLEM DESCRIPTION:** Interface elements must clearly indicate their function so that the user has clues about their use. The materials should be identified in the plural (tiles, floors...), because in the singular they do not indicate to the user that there are options to choose from if they click on a certain type of material.
**SOLUTION PROPOSAL:** Correct designation of materials, putting them in the plural
**SEVERITY DEGREE:** 1
**Image of the interface**

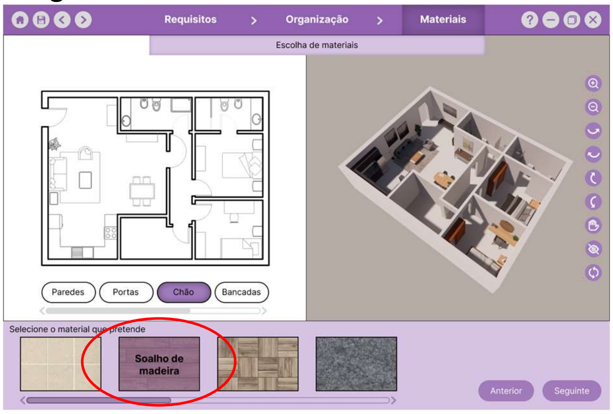

Delete walls

**PROBLEM DESIGNATION:** Delete walls
**HEURISTICS VIOLATED:** 2- Match between the system and the real world (Nielsen's Heuristics)
**PROBLEM DESCRIPTION:** It lacks relating the action to what would happen in the real world. When eliminating walls, the system must simulate what will happen to that wall.
**SOLUTION PROPOSAL:** Place the icon of an eraser, or a hammer for the person to erase (or destroy) the wall directly on the floorplan
**SEVERITY DEGREE:** 1
**Image of the interface**

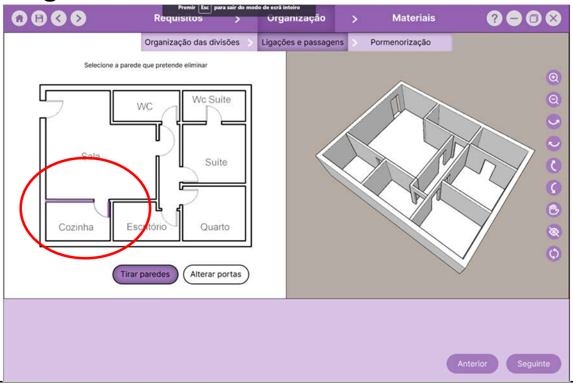

Windows

**PROBLEM DESIGNATION:** Windows
**HEURISTICS VIOLATED:** 2- Match between the system and the real world (Nielsen's Heuristics)
**PROBLEM DESCRIPTION:** Windows should be represented, as this may affect the choice of, e.g., the kitchen layout.
**SOLUTION PROPOSAL:** Change the representation of the architectural project
**SEVERITY DEGREE:** 1
**Image of the interface**

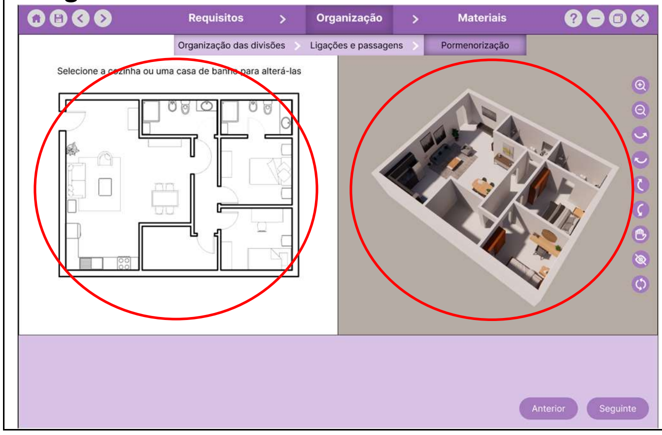

Supplement: S3 File — (PDF) [file pone.0313291.s003.pdf]
