# Supplementary material for: Bridging the gap in customised housing design: Integrating a graphic user interface for user collaboration
Source: PLoS One. 2024 Dec 20;19(12):e0313291. doi: 10.1371/journal.pone.0313291 (PMC11661643; doi:10.1371/journal.pone.0313291)
Supplement: S5 File — (PDF) [file pone.0313291.s005.pdf]

Tasks script

Imagine that you have recently joined a housing cooperative and an apartment building is going to be built. You will use this system to customise your house.

Imagine that the choices indicated in the tasks below would be the options you prefer and therefore you chose them. Also, imagine that you want a house for a household of **3 people**. The choice of the house is made by member order, so there may be unavailable options to choose from.

Tasks to be performed by the participant:

➔ **Task 1:** Log in with the following access data:
Email: exemplo@codesign.pt
Password: participar

➔ **Task 2:** Imagine that you are going to choose your house, and you choose apartment 1D (T2), on the 1st floor, in the project at Rua Cristóvão Rodrigues Acenheiro.

➔ **Task 3:**

- Imagine you want to have in your house a kitchen with the size **S**
- Imagine you want to have in your house a bedroom for one person with the size **S**
- Imagine you want to have in your house a bathroom with the size **S**
- Imagine you want to have in your house a living room with the size **M**
- Imagine you want to have in your house a suite for two persons with the size **S**

➔ **Task 4:** Imagine that, besides the available rooms, you want to add a customized room with the name "office" and that it has the size **S**.
Go to the next phase

➔ **Task 5:** Imagine that the option you liked the most was option 3. Choose it and customise it by switching the bedroom and the suite.
Go to the next phase

➔ **Task 6:** Imagine that you didn't like the position of the door between the living room and the hallway. Change it to near the toilet. Imagine that you are satisfied with the size of the door, so keep it with the S size.

➔ **Task 7:** Imagine that you want to remove the wall between the living room and the kitchen. Remove the wall to connect these spaces.
Go to the next phase

➔ **Task 8:**

- Imagine that you want to change the kitchen format for a "U" kitchen
- Imagine that you want to change the layout of the bathroom in the suite and you liked the first option.
- Imagine that you want to change the layout of the bathroom that connects to the hallway and that you liked the third option.

Go to the next phase

➔ **Task 9:** Imagine that you want to put a wooden floor on the floor of your living room and you liked the first type of wooden floor

➔ **Task 10:** Imagine that you have already chosen all the materials for your home. Finalize the process of customizing your home and then submit the project
