# Supplementary material for: Bridging the gap in customised housing design: Integrating a graphic user interface for user collaboration
Source: PLoS One. 2024 Dec 20;19(12):e0313291. doi: 10.1371/journal.pone.0313291 (PMC11661643; doi:10.1371/journal.pone.0313291)
Supplement: S6 File — (PDF) [file pone.0313291.s006.pdf]

Preliminary questionnaire

Participant number:

1. **Age**
   ☐ 25 – 30
   ☐ 31 – 35
   ☐ 36 – 40
   ☐ 41 – 45

2. **Gender**
   ☐ Male
   ☐ Female
   ☐ Other (Please, specify) ____

3. **What is your education level?**
   ☐ Until 9th grade
   ☐ Between 9th and 12th grade
   ☐ Higher education

4. **What is your profession /training?**

   ____

5. **How do you evaluate your relationship with digital technologies?**
   ☐ Very bad
   ☐ Insufficient
   ☐ Reasonable
   ☐ Good
   ☐ Very Good

6. **Have you ever had contact with architectural projects?**
   ☐ Sim
   ☐ Não

7. **At this moment I feel… (check those that apply)**
   ☐ Motivated
   ☐ Apprehensive
   ☐ Other (Please, specify) ____

Thank you for your participation!

Single Ease Question Questionnaire

Participant number:

Please answer each question after completing each task.

Task 1

Overall, how easy or difficult was it to accomplish this task?

Very difficult						Very easy
1	2	3	4	5	6	7

Task 2

Overall, how easy or difficult was it to accomplish this task?

Very difficult						Very easy
1	2	3	4	5	6	7

Task 3

Overall, how easy or difficult was it to accomplish this task?

Very difficult						Very easy
1	2	3	4	5	6	7

Task 4

Overall, how easy or difficult was it to accomplish this task?

Very difficult						Very easy
1	2	3	4	5	6	7

Task 5

Overall, how easy or difficult was it to accomplish this task?

Very difficult						Very easy
1	2	3	4	5	6	7

Task 6

Overall, how easy or difficult was it to accomplish this task?

Very difficult						Very easy
1	2	3	4	5	6	7

Task 7

Overall, how easy or difficult was it to accomplish this task?

Very difficult						Very easy
1	2	3	4	5	6	7

Task 8

Overall, how easy or difficult was it to accomplish this task?

Very difficult						Very easy
1	2	3	4	5	6	7

Task 9

Overall, how easy or difficult was it to accomplish this task?

Very difficult						Very easy
1	2	3	4	5	6	7

Task 10

Overall, how easy or difficult was it to accomplish this task?

Very difficult						Very easy
1	2	3	4	5	6	7

Thank you for your participation!

Sistem Usability Scale Questionnaire

Participant number:

For each of the statements below, select the box (only one per line) that best describes your opinion about the tool you just tried:

	Totally disagree				Totally Agree
1- I think that I would like to use this system frequently	1	2	3	4	5
2- I found the system unnecessarily complex	1	2	3	4	5
3- I thought the system was easy to use	1	2	3	4	5
4- I think that I would need the support of a technical person to be able to use this system	1	2	3	4	5
5- I found the various functions in this system were well integrated	1	2	3	4	5
6- I thought there was too much inconsistency in this system	1	2	3	4	5
7- I would imagine that most people would learn to use this system very quickly	1	2	3	4	5
8- I found the system very cumbersome to use	1	2	3	4	5
9- I felt very confident using the system	1	2	3	4	5
10- I needed to learn a lot of things before I could get going with this system	1	2	3	4	5

Thank you for your participation!

Graphical User Interface (GUI) Questionnaire

Participant number:

For each of the statements below, select the box (only one per line) that best describes your opinion about the tool you just tried:

	Totally disagree 1	2	3	4	Totally Agree 5
1- I understood the names of the design phases.					
2- It's not easy to navigate through the menus.					
3- I understood the menu options and instructions.					
4- Menu icons and buttons do not clearly indicate their functionality.					
5- Graphics and diagrams are easy to read and understand.					
6- The screen graphical design is not well designed.					
7- The information is well organized on the screens.					
8- Não é fácil aceder às informações de que preciso					
9- The size of the buttons and words or statements is appropriate.					

Thank you for your participation!
