# Supplementary material for: Bridging the gap in customised housing design: Integrating a graphic user interface for user collaboration
Source: PLoS One. 2024 Dec 20;19(12):e0313291. doi: 10.1371/journal.pone.0313291 (PMC11661643; doi:10.1371/journal.pone.0313291)
Supplement: Attachment — Submitted filename: Response to Academic Editor.docx [file pone.0313291.s009.docx]

**Response to reviewers**

| **Response to Academic editor** | |
| --- | --- |
| The authors will ensure to provide a link to the complete dataset. | The link was provided in the submission form in the Data Availability Statement tab, through the following statement:  “All Supporting Information files (S1_File: Results of the heuristic evaluation, S2_File:  Description of each usability issue, S3_File: Results of the summative evaluation with  inhabitants, S4_File: Tasks script inhabitants followed in the summative evaluation,  S5_File: Questionnaires applied in the summative evaluation, S6_File: Results of the discussion with architects) are available from the Zenodo database (accession number(s) 10.5281/zenodo.12187614).”  We created a new version with additional Supporting Information files as requested, and the statement was updated with the new Zenodo link ([10.5281/zenodo.12187613](https://zenodo.org/doi/10.5281/zenodo.12187613)).  We did not include this statement in the manuscript to preserve anonymity for blind review. This was explained in the Supporting Information section of the manuscript. The link will be included in the manuscript as soon as we can disclose the authors' identities. |
| The scope of the study needs to be more clearly articulated in the introduction and in the abstract and title. It appears that the focus of the manuscript is that of designing, developing, prototyping and testing a graphical user interface that facilitates end-user participation in co-designing buildings. | We appreciate the comment.  We improved the introduction and abstract to accomodate the reviewer critic.  We also revised the title, to clarify the paper’s purpose |
| The framework needs to be enriched by including further reference to generative design tools and a discussion on the challenges when engaging users in participatory design. | We have further developed the framework section, including references suggested by the reviewer, which address the topics mentioned. |
| In the methodology, a clearer justification for the choice of the sample could be provided by indicating the criteria used to select the professionals and housing cooperatives for the study. For example why were there 15 professionals from Portugal and 13 from other countries, how were the latter identified and selected for the study? | In the methodology section, we clarified the selection criteria of participants from each group of interviewees. We also explained that we balanced the number of national and foreign professionals interviewed. Additionally, we clarified the selection of participants for the different iterations of the prototype evaluation. |
| In the methodology, it would be helpful to include a diagram summarizing the 8 steps and how these feed into the development, prototyping, testing and evaluation of the user interface. It would be helpful to provide the interview protocols used with the three groups of interviewees. | Thank you for the suggestion. We included the diagram in the methods section, as suggested, and revised the text related to the steps.  We provided the interview protocols used with three groups of interviewees by including them in the dataset hosted at Zenodo. The updated link was provided through the Data Availability Statement in the submission platform and will be included in the manuscript as soon as the authors identities can be disclosed. |
| The presentation of the manuscript needs to be improved. For example, the section titled: “Collection of user needs by interviews – step 1’ is somewhat confusing as some of the headings refer to “interview” outcomes and other subheading refer to the “steps” used to design the digital interface.  It would be good to be consistent and refer to the steps in the design and use the findings from the interviews with the 3 groups of respondents to explain how these findings were used to inform each of the 8 steps. The section is too long and the authors could use sub-headings to explain the steps involved and then include a separate section tackling user perceptions and feedback. On p. 20, which step does ‘Evaluation of the high-fidelity prototype’ refer to ? | Thank you for the comment.  The referred section includes only subheadings that refer to the interview outcomes. The section “Definition of the system” refers to the second, third and fourth steps and is a first-heading section.  Nevertheless, for the remaining steps, we have revised the headings structure so that sections directly correspond to the steps of the iterative process explained in the methodology. Sections with subheadings identify the steps included. |
| The discussion is very descriptive and though it provides useful practical tips on GUI and their applications, there is very limited (if any) discussion on how the study engages with and builds on relevant literature on the use of GUI in co-design and participatory design. The reviewer's comments below provide valuable insights on how this could be improved. | Thank you for the comment. In the discussion, we revised the text regarding this aspect, by adding that the user-centred design methodology used in this research is not usually used in the development of graphical user interfaces for co-design, as far as we could find in the literature. There are authors who tested GUIs but did not involve end-users in the GUI development process from early phases or in an iterative way. Our work follows this methodology as it is considered a paramount way of achieving a product that meets end-users needs and satisfaction in GUIs development |
| The authors need to acknowledge that the manuscript is based on work presented and published at the 'International Conferences Interfaces and Human Computer Interaction' in 2023. | Thank you for the comment. We added an anonymised sentence in the Acknowledgement section. As soon as we can disclose the author’s identity, we will replace the sentence to include such information. |
