# Supplementary material for: Bridging the gap in customised housing design: Integrating a graphic user interface for user collaboration
Source: PLoS One. 2024 Dec 20;19(12):e0313291. doi: 10.1371/journal.pone.0313291 (PMC11661643; doi:10.1371/journal.pone.0313291)
Supplement: Attachment — Submitted filename: Response to reviewers.docx [file pone.0313291.s010.docx]

Dear Editor of PLOS ONE,

We are pleased to submit the revised version of the Manuscript, addressing the minor revisions required.

As suggested, we amended the title and corrected all the points raised in the decision letter. Additionally, we corrected a sentence in the methodology (p.14, Line 308) by substituting “steps 8 and 9” for “steps 9 and 10”, as we detected it was not correct as it was. Some punctuation issues we identifyed were also corrected.

We also have disclosed all the information previously omitted for blind review:

(1) We added the authors' names and affiliations on the title page
(2) We disclosed the name of the institution that provided the Ethics approvement
(3) We added the in-text references to our own work, which were previously omitted throughout the text
(4) We acknowledged that this work is based on the work published and presented at the IHCI 2023 International Conference, on the acknowledgement section
(5) We provided in the Supporting Information section the link for the repository (Zenodo) that hosts the dataset

Thank you for your time and consideration.

Sincerely,

Micaela Raposo, Sara Eloy and Miguel Sales Dias
